# Tobacco usage in the home: a cross-sectional analysis of heated tobacco product (HTP) use and combustible tobacco smoking in Japan, 2023

**DOI:** 10.1265/ehpm.23-00292

**Published:** 2024-03-05

**Authors:** Satomi Odani, Takahiro Tabuchi

**Affiliations:** 1 Graduate School of Medicine, Osaka University, 2-2 Yamadaoka, Suita, Osaka 565-0871, Japan; 2 The Tokyo Foundation for Policy Research, 3-2-1 Roppongi, Minato-ku, Tokyo 106-5234, Japan; 3 Cancer Control Center, Osaka International Cancer Institute, 3-1-69 Otemae Chuo-ku, Osaka, Osaka 540-0008, Japan

**Keywords:** Heated tobacco product, Internet survey, Secondhand smoking, Smoke-free environment, Tobacco control, Tobacco use prevalence

## Abstract

**Background:**

Heated tobacco product (HTP) use continues in Japan as the second most common product after cigarettes. While the health effects of HTPs and their secondhand emissions are not well-studied, the tobacco industry has actively marketed HTPs as a smokeless, health-conscious alternative to cigarettes to encourage home consumption. We investigated the prevalence of current tobacco product use and usage at home.

**Methods:**

The present study conducted a cross-sectional analysis of data from the 2023 wave of a nationwide, Internet-based, self-reported survey. 29,354 individuals aged 16–74 were included in the analysis. We assessed the prevalence of current (past-30-day) use for HTPs, cigarettes, non-cigarette combustible tobacco, and dual (combustible plus HTP) use. The frequency of use (daily or more than monthly) in the home was calculated for both HTPs and combustible tobacco. Multivariable Poisson regression models were employed to identify factors associated with home usage. Adjusted prevalence ratios (APRs) and 95% confidence intervals (CIs) were computed. All analyses were weighted to address the Internet-based sample's selectivity and yield nationally representative estimates.

**Results:**

In 2023, the prevalence of current use was 12.4% (HTPs), 18.9% (cigarettes), 3.6% (non-cigarette combustible tobacco), and 7.4% (dual use). Among current users of any tobacco (N = 5,818), 49.8% reported daily tobacco usage within their homes, and 67.1% reported monthly or more frequent home usage. Compared to exclusive combustible tobacco smokers, exclusive HTP users exhibited higher prevalence of daily home usage (APR = 1.54; 95% CI = 1.43–1.67), as did dual users (APR = 1.10; 95% CI = 1.01–1.20). Daily home usage prevalence was notably higher for those without complete tobacco-free rules at home or workplaces, older individuals, and those with lower education levels. Those living with adult or child household member and current drinkers showed significantly lower daily home usage prevalence.

**Conclusion:**

Home usage was more common among HTP users than among combustible tobacco smokers. Ongoing efforts to assess and address the impact of indoor tobacco product use, including HTPs, on health are warranted. Regulatory and educational strategies should be considered to discourage tobacco consumption in both public and private spaces.

**Supplementary information:**

The online version contains supplementary material available at https://doi.org/10.1265/ehpm.23-00292.

## Introduction

Japan features a distinctive tobacco product landscape marked by the notable prevalence of heated tobacco products (HTPs), which have emerged as the second most common product, following conventional cigarettes [1]. The prevalence of HTP use has consistently remained high, hovering around 11–12% since 2019 [1–3].

While the health effects of HTPs and their secondhand emissions remain inadequately studied, the tobacco industry has actively positioned HTPs as “reduced-exposure” alternatives [4–6]. This positioning asserts that HTPs offer a health-conscious choice for individuals concerned about exposing others to secondhand smoke (SHS) generated by conventional cigarettes. Notably, this marketing approach intensified during the COVID-19 pandemic, as the industry employed newspaper and online advertisements to encourage HTP usage at home, especially during periods when government-recommended stay-at-home directives were in place [7, 8].

In the legislative domain, the revised Health Promotion Act, sanctioned in July 2018 and progressively implemented by April 1, 2020, prohibits all forms of tobacco use within specific public domains such as government offices, healthcare facilities, and educational institutions [9]. Concurrently, it permits outdoor tobacco use spaces on premises. The Act encompasses indoor tobacco use restrictions across a diverse array of public settings, including workplaces, restaurants, cafes, and bars [9]. These restrictions, however, allow provisions for designated smoking rooms, as well as specific areas designated for the use of HTPs where patrons can also partake in food and beverage consumption. Notably, the Act’s jurisdiction does not extend to private spaces such as homes. Consequently, the implementation of tobacco-free regulations within these domains relies on voluntary initiatives.

In Japan, workplaces and homes stand as the two primary sources of exposure to SHS and secondhand aerosol from HTPs [10, 11]. Previous research has highlighted that smoke-free workplaces not only shield non-smokers from the hazards of secondhand smoking but also foster an environment that encourages smokers to quit or reduce tobacco consumption [12, 13]. The recently revised Health Promotion Act is expected to amplify this effect by ensuring tobacco-free environments in indoor public places. However, given the challenge of legally restricting tobacco use in private spaces, concerns arise regarding the surging popularity of HTPs in Japan. Coupled with the health-conscious image of HTPs not exposing others to harmful combusted tobacco smoke, these factors may have undermined efforts to denormalize tobacco use in such settings.

Considering these factors, the objectives of this study are to assess the most recent prevalence of tobacco product use in Japan and examine the prevalence and underlying factors of tobacco product use within homes. Of particular interest is the exploration of differences in home tobacco use, contingent upon the specific type of product used, namely HTPs or combustible tobacco.

## Methods

### Data source

The present study employed a cross-sectional analysis of the 2023 wave of the Japan Society and New Tobacco Internet Survey (JASTIS). This nationwide survey utilized an internet-based platform and relied on self-reported data. A random selection of participants was drawn from a pool of over two million panelists provided by a private vendor, Rakuten Insight Inc [14]. The selection process considered various demographic and socioeconomic factors, such as education, housing, and marital status, as defined by the Japan census. Data collection took place from February 6 to February 27, 2023. To ensure data quality, a total of 2,963 individuals were excluded due to providing unreasonable responses. Unreasonable responses included consistently providing the same answer across a set of questions, selecting all multiple-choice options for questions regarding illegal substance use or chronic conditions, and choosing an incorrect response to a validation question [15]. Additionally, individuals aged 75 years or older (1,683 individuals) were excluded to maintain consistency with a previous report [1], resulting in a final sample size of 29,354 individuals for the analysis. Further details regarding the sampling methods can be found elsewhere [15]. The study received ethical approval from the Research Ethics Committee of the Osaka International Cancer Institute (approval number 20094-2).

### Prevalence of current tobacco product use

The prevalence of current tobacco product use was assessed by considering the past 30-day usage of various tobacco products, including heated tobacco products (HTPs), manufactured and roll-your-own cigarettes, non-cigarette combustible tobacco products (little cigars, pipes, and water pipes), as well as dual use of HTPs and combustible tobacco. Those who reported using a specific tobacco product on one or more days within the past 30 days were classified as current users of that product. The questionnaire provided a list of product names and dedicated answer spaces for each tobacco product. Additionally, specific brand examples were given for HTPs (Ploom Tech, Ploom S, Ploom X, IQOS, glo, and lil HYBRID) to enhance clarity and improve the accuracy of respondents in answering survey questions. These product-specific questions were considered to possess a high level of face and construct validity for accurately identifying current tobacco product users.

### Tobacco product use in the home

Respondents were asked about the frequency of tobacco product use in their homes. The response categories included “every day,” “several days a week,” “about once a week,” “about once a month,” “none,” and “I did not visit this place.” Separate questions were used to gather data on both HTP use and combustible tobacco smoking. Dichotomous variables were separately created to assess daily use and use at least once a month (≥monthly) in the home. We specifically examined HTP use among current HTP users, combustible tobacco smoking among current smokers, and any tobacco product use among current users of any tobacco product. Respondents who had not stayed in their homes in the past month were excluded from the analysis. This exclusion led to the removal of 275 and 539 individuals among current HTP users and combustible tobacco smokers, respectively. Consequently, the analysis included 3,082, 4,769, and 5,818 individuals for current HTP users, combustible tobacco smokers, and any tobacco users, respectively.

### Covariate

Sex, age, home ownership, education (categorized as high school or below, or beyond high school [including vocational school, 2-year/4-year college]), alcohol use, and whether they cohabitated with adults or child/children under 19 years of age were evaluated. Current tobacco product users were categorized based on the type of product used, which included combustible tobacco only, HTP only, or dual use. Additionally, participants were asked about the tobacco use rule in their homes through the following question: “Which statement best describes the rules about using tobacco products inside your home?” The response options were: “Neither combustible tobacco nor HTPs allowed,” “Both combustible tobacco and HTPs allowed,” “Only HTPs allowed,” “Only combustible tobacco allowed,” “Not applicable (e.g. being institutionalized),” and “Don’t know.” The response “Neither combustible tobacco nor HTPs allowed” was considered indicative of a complete tobacco-free home rule. Furthermore, the study assessed the tobacco use policy in the workplace by inquiring about whether tobacco use was permitted inside or outside the workplace, allowed only in indoor designated areas, allowed anywhere on the premises, or if respondents were unaware of the policy, or if this question were not applicable due to current unemployment.

### Statistical analysis

Descriptive analyses were conducted to determine the prevalence of tobacco product use and the percentage of current tobacco product users who used the product in their homes. Multivariable Poisson regression was employed to investigate the factors associated with daily and ≥monthly tobacco product use in the home. Adjusted prevalence ratios (APRs) and their corresponding 95% confidence intervals (CIs) were calculated by controlling for all the aforementioned characteristics. All analyses were weighted using the inverse probability weight (IPW) to address the selectivity of the internet-based sample. To obtain the IPW, propensity scores for “being an internet survey respondent” were computed through logistic regression models, which were adjusted for basic demographic, socioeconomic, health-related, and tobacco-use-related factors between the 2023 JASTIS sample and a nationally representative sample [16]. Further details regarding the weighting procedure can be found elsewhere [15, 17]. Statistical analyses were performed using R version 4.1.3.

## Results

### Prevalence of current tobacco product use

In 2023, the prevalence of current tobacco product use (past 30-day use) was: 12.4% for HTPs, 18.9% for cigarettes, 3.6% for non-cigarette combustible tobacco, and 7.4% for dual tobacco product use (Table 1). 24.5% of respondents reported using at least one specific tobacco product in the past 30 days. Among the evaluated non-cigarette combustible tobacco products, the prevalence of smoking little cigars, pipes, and water pipes were each 2.1% (data not shown). Overall, 46.7% of respondents reported implementing a complete tobacco-free rule within their homes, while 23.6% reported partial or no tobacco-free rules, and 29.8% indicated a lack of awareness or applicability of the rule. Adoption of a complete tobacco-free home rule corresponded to lower tobacco use prevalence (14.9%), while prevalence was notably higher for those with partial (66.9% among those allowing HTPs, 47.4% among those allowing combustible tobacco smoking) or no rules (53.9%). Respondents reporting a complete tobacco-free policy in their workplaces exhibited a 22.7% prevalence of current tobacco use, in contrast to a prevalence of 44.8% among those with unrestricted tobacco use in their workplaces. The prevalence of any current tobacco product use was higher among men compared to women, among current drinkers compared to non-current/never drinkers, and lower among individuals aged 16–19 and those aged 60 and above, across all assessed tobacco products. Individuals with lower educational attainment (high school or below) exhibited higher prevalence for all tobacco products except non-cigarette combustible tobacco. Additionally, among respondents living with child/children, 27.7% were current users of any tobacco product. Refer to Supplementary Table 1 for stratified prevalence of current tobacco use based on sex.

**Table 1:** Prevalence of current tobacco product use in Japan, 2023

	**Distribution**	**Prevalence**				

		**Heated ** **tobacco ** **products**	**Cigarettes**	**Non-cigarette combustible ** **tobacco ** **products**	**Dual ** **(combustible + ** **heated tobacco) ** **use**	**Any (1+) ** **tobacco ** **product**

	**N (%)**	**% (SE)**	**% (SE)**	**% (SE)**	**% (SE)**	**% (SE)**
Overall	29354 (100.0%)	12.4 (0.3)	18.9 (0.4)	3.6 (0.2)	7.4 (0.2)	24.5 (0.4)
Home tobacco use rule						
No tobacco allowed	14712 (46.7%)	5.5 (0.3)	12.0 (0.4)	1.8 (0.2)	3.0 (0.2)	14.9 (0.4)
Only HTPs allowed	1452 (5.7%)	59.2 (1.9)	35.5 (1.8)	10.9 (1.2)	28.6 (1.7)	66.9 (1.9)
Only combustible tobacco allowed	323 (1.1%)	34.3 (3.8)	36.4 (3.8)	20.1 (3.0)	25.7 (3.3)	47.4 (4.0)
Any tobacco allowed	4773 (16.8%)	25.3 (0.9)	44.6 (1.1)	6.5 (0.5)	17.1 (0.8)	53.9 (1.1)
Don’t know/Not applicable	8094 (29.8%)	6.2 (0.4)	11.4 (0.6)	2.9 (0.3)	4.1 (0.4)	14.2 (0.6)
Workplace tobacco use policy						
No tobacco allowed	8283 (25.3%)	12.6 (0.6)	18.0 (0.6)	5.2 (0.4)	8.6 (0.5)	22.7 (0.7)
Allowed, outdoor only	6942 (24.5%)	15.9 (0.6)	23.1 (0.8)	3.2 (0.3)	8.3 (0.5)	31.5 (0.8)
Allowed, in designated indoor spaces only	2979 (9.8%)	22.8 (1.2)	29.0 (1.3)	6.2 (0.7)	13.5 (1.0)	39.1 (1.4)
Allowed, anywhere	580 (2.4%)	22.0 (2.4)	33.7 (2.7)	3.9 (0.8)	12.2 (1.8)	44.8 (3.0)
Don’t know/Not applicable	10570 (37.9%)	6.7 (0.4)	13.1 (0.5)	2.2 (0.2)	4.2 (0.3)	16.2 (0.6)
Sex						
Man	14482 (49.2%)	18.9 (0.5)	27.6 (0.6)	5.4 (0.3)	11.6 (0.4)	35.8 (0.6)
Woman	14872 (50.8%)	6.1 (0.3)	10.4 (0.4)	1.9 (0.2)	3.4 (0.2)	13.6 (0.4)
Age, years						
16–19	722 (2.5%)	7.0 (1.5)	6.8 (1.4)	4.6 (1.2)	5.5 (1.3)	9.2 (1.6)
20–29	5487 (18.8%)	14.7 (0.8)	16.8 (0.8)	7.0 (0.5)	11.1 (0.7)	21.7 (0.9)
30–39	5752 (19.7%)	16.3 (0.7)	18.7 (0.8)	4.0 (0.4)	9.4 (0.6)	26.3 (0.9)
40–49	5485 (18.8%)	16.4 (0.7)	23.4 (0.8)	3.5 (0.4)	8.8 (0.6)	31.4 (0.9)
50–59	4573 (15.6%)	10.7 (0.6)	20.6 (0.8)	2.0 (0.3)	5.5 (0.4)	26.2 (0.9)
60–74	7335 (24.7%)	6.2 (0.4)	17.2 (0.7)	1.7 (0.2)	3.4 (0.3)	20.5 (0.8)
Home ownership						
No	10288 (26.4%)	13.6 (0.5)	20.1 (0.6)	4.7 (0.3)	8.3 (0.4)	26.3 (0.6)
Yes	19066 (73.6%)	12.0 (0.4)	18.4 (0.4)	3.2 (0.2)	7.1 (0.3)	23.9 (0.5)
Household members						
Alone	6647 (17.8%)	13.7 (0.7)	20.8 (0.8)	5.3 (0.4)	8.9 (0.6)	26.5 (0.8)
Adults only	13913 (50.6%)	9.6 (0.4)	17.3 (0.5)	2.7 (0.2)	5.6 (0.3)	21.8 (0.5)
Child/children present	8794 (31.6%)	16.2 (0.6)	20.3 (0.6)	4.2 (0.3)	9.4 (0.5)	27.7 (0.7)
Education						
High school or below	7846 (53.9%)	13.3 (0.5)	21.8 (0.6)	3.4 (0.3)	7.8 (0.4)	27.8 (0.6)
Beyond high school	21276 (46.1%)	11.5 (0.3)	15.6 (0.3)	3.7 (0.2)	6.9 (0.2)	20.9 (0.4)
Alcohol use						
Non-current/never	13110 (46.5%)	7.4 (0.3)	12.8 (0.5)	1.6 (0.1)	3.9 (0.3)	16.7 (0.5)
Current	16244 (53.5%)	16.8 (0.4)	24.1 (0.5)	5.4 (0.3)	10.5 (0.4)	31.3 (0.5)

**Abbreviation:** HTP = heated tobacco product, SE = standard error.

**Note:** Data were weighted to address the selectivity of the internet-based sample using a nationally representative sample of Japanese population.

1. Cigarettes assessed in this study included manufactured cigarettes and roll-your-own cigarettes

2. Non-cigarette combustible tobacco products assessed in this study included little cigars, pipes, and water pipes

3. Heated tobacco products (HTPs) assessed in this study included Ploom Tech, Ploom S, Ploom X, IQOS, glo, and lil HYBRID

### Tobacco product use in the home

Among current users of HTPs (N = 3,082), 52.2% reported daily use of the product within their homes, and among current smokers of combustible tobacco products (N = 4,769), this figure was 38.2% (Table 2). These percentages varied significantly based on adoption of tobacco-free home rules, with the lowest percentages observed among those reporting a complete tobacco-free home rules (20.0% for daily HTP use, 10.2% for combustible tobacco smoking). The highest percentage of daily home usage of HTP was noted among those with specific home rules allowing HTP use (71.8%), while the highest percentage of daily home smoking of combustible tobacco was among those without any tobacco-free home rules (63.8%). Furthermore, 78.4% of HTP users and 57.9% of combustible tobacco smokers reported using the respective products in their homes at least once a month.

**Table 2:** Tobacco product use in the home among current HTP users and combustible tobacco smokers

	**Distribution**		**Daily use in the home**		**≥Monthly use in the home**	

	**Current HTP users**	**Current combustible tobacco ** **smokers**	**HTP use ** **(among current HTP users, N = 3082)**	**Combustible tobacco smoking (among current smokers, N = 4769)**	**HTP use ** **(among current HTP users, N = 3082)**	**Combustible tobacco smoking (among current smokers, N = 4769)**

	**N (%)**	**N (%)**	**% (SE)**	**% (SE)**	**% (SE)**	**% (SE)**
Overall	3082 (100.0%)	4769 (100.0%)	52.2 (1.3)	38.2 (1.0)	78.4 (1.0)	57.9 (1.1)
Home tobacco use rule						
No tobacco allowed	655 (19.6%)	1410 (27.8%)	20.0 (2.1)	10.2 (1.2)	38.5 (2.6)	20.5 (1.5)
Only HTPs allowed	821 (28.5%)	492 (11.1%)	71.8 (2.3)	22.1 (2.4)	94.7 (1.0)	59.2 (3.2)
Only combustible tobacco allowed	93 (2.7%)	120 (2.2%)	37.1 (7.3)	25.2 (5.7)	80.3 (6.7)	76.4 (5.0)
Any tobacco allowed	1124 (36.0%)	2080 (42.7%)	58.5 (2.2)	63.8 (1.6)	88.1 (1.4)	83.2 (1.2)
Don’t know/Not applicable	389 (13.2%)	667 (16.2%)	43.5 (3.7)	31.9 (2.7)	75.7 (3.1)	52.1 (2.9)
Workplace tobacco use policy						
No tobacco allowed	857 (26.2%)	1274 (24.4%)	41.4 (2.4)	29.4 (1.8)	79.9 (1.9)	61.3 (2.0)
Allowed, outdoor only	939 (31.8%)	1389 (30.5%)	53.7 (2.2)	34.7 (1.8)	73.0 (2.0)	49.4 (1.9)
Allowed, in designated indoor spaces only	570 (18.7%)	731 (15.7%)	55.0 (3.0)	40.1 (2.7)	80.6 (2.2)	60.5 (2.6)
Allowed, anywhere	126 (4.3%)	215 (4.5%)	75.5 (5.0)	55.8 (4.6)	91.8 (2.3)	70.7 (4.2)
Don’t know/Not applicable	590 (18.9%)	1160 (24.9%)	56.5 (3.1)	47.0 (2.2)	80.3 (2.2)	61.1 (2.1)
Sex						
Man	2276 (75.2%)	3355 (72.1%)	50.4 (1.5)	36.8 (1.2)	77.2 (1.2)	58.4 (1.2)
Woman	806 (24.8%)	1414 (27.9%)	57.7 (2.7)	42.1 (2.0)	82.2 (1.9)	56.8 (2.1)
Age, years						
16–19	38 (1.2%)	49 (0.9%)	18.8 (9.6)	8.5 (6.3)	81.5 (11.5)	70.2 (8.7)
20–29	685 (22.0%)	807 (17.3%)	36.1 (2.9)	20.0 (2.2)	80.2 (2.2)	60.1 (2.8)
30–39	679 (25.4%)	796 (19.6%)	48.2 (2.6)	27.3 (2.2)	74.6 (2.2)	49.9 (2.5)
40–49	719 (25.1%)	992 (22.5%)	57.6 (2.5)	40.6 (2.1)	76.2 (2.1)	56.9 (2.1)
50–59	490 (13.7%)	918 (17.3%)	68.5 (2.7)	50.0 (2.2)	84.1 (2.1)	59.5 (2.1)
60–74	471 (12.6%)	1207 (22.3%)	62.9 (3.4)	51.7 (2.3)	81.0 (2.5)	62.6 (2.2)
Education						
High school or below	945 (58.8%)	1552 (61.7%)	55.6 (2.0)	42.1 (1.5)	80.0 (1.6)	60.0 (1.6)
Beyond high school	2115 (41.2%)	3184 (38.3%)	47.5 (1.4)	31.9 (1.1)	75.8 (1.2)	54.3 (1.1)
Home ownership						
No	1198 (28.6%)	1750 (28.3%)	51.9 (2.0)	40.4 (1.7)	79.8 (1.7)	61.0 (1.6)
Yes	1884 (71.4%)	3019 (71.7%)	52.3 (1.6)	37.4 (1.3)	77.9 (1.3)	56.7 (1.3)
Household members						
Alone	769 (19.3%)	1244 (20.2%)	55.7 (2.7)	45.3 (2.1)	88.8 (1.6)	70.3 (1.9)
Adults only	1202 (39.7%)	2150 (46.4%)	57.2 (2.1)	43.9 (1.6)	79.7 (1.6)	59.5 (1.6)
Child/Children present	1111 (40.9%)	1375 (33.4%)	45.6 (2.1)	26.1 (1.6)	72.3 (1.8)	48.2 (1.9)
Alcohol use						
Non-current/never	779 (27.3%)	1337 (30.9%)	61.7 (2.4)	46.0 (2.0)	78.9 (2.0)	59.8 (2.0)
Current	2303 (72.7%)	3432 (69.1%)	48.6 (1.5)	34.8 (1.2)	78.2 (1.2)	57.1 (1.2)
Current HTP use						
No	-	2875 (60.2%)	-	43.1 (1.3)	-	51.3 (1.4)
Yes	-	1894 (39.8%)	-	31.0 (1.5)	-	68.0 (1.6)
Current combustible tobacco smoking						
No	1216 (40.8%)	-	69.1 (1.8)	-	78.8 (1.6)	-
Yes	1866 (59.2%)	-	40.5 (1.6)	-	78.2 (1.3)	-

**Abbreviation:** HTP = heated tobacco product, SE = standard error.

**Note:** Data were weighted to address the selectivity of the internet-based sample using a nationally representative sample of Japanese population. Respondents who had not stayed in their homes in the past month were excluded from the analysis (275 and 539 individuals among current HTP users and combustible tobacco smokers, respectively).

1. Combustible tobacco products assessed in this study included cigarettes (manufactured and roll-your-own cigarettes), little cigars, pipes, and water pipes

2. Heated tobacco products (HTPs) assessed in this study included Ploom Tech, Ploom S, Ploom X, IQOS, glo, and lil HYBRID

Among all current tobacco product users (N = 5,818), 49.8% reported daily tobacco use within their homes (Table 3). Notably, exclusive HTP users exhibited a higher likelihood of daily home use (Adjusted Prevalence Ratio [APR] = 1.54; 95% Confidence Interval [CI] = 1.43–1.67) compared to exclusive combustible tobacco smokers. Similarly, dual users had an elevated likelihood of daily home use (APR = 1.10; 95%CI = 1.01–1.20). Tobacco use home rules were significantly associated with daily home usage, with the highest likelihood observed among those without any tobacco-free home rules (APR = 4.57; 95%CI = 3.83–5.46), followed by those allowing HTP use (APR = 4.02; 95%CI = 3.33–4.86) and those allowing combustible tobacco smoking (APR = 3.02; 95%CI = 2.19–4.18). Participants reporting incomplete or absent tobacco-free policies inside their workplaces also exhibited increased likelihoods of daily home tobacco use (APR = 1.17; 95%CI = 1.06–1.31 and APR = 1.28; 95%CI = 1.13–1.45, respectively) compared to those with complete workplace tobacco-free policies. Factors such as older age, particularly among those aged 50–59 years (APR = 1.64; 95%CI = 1.42–1.90, compared to those aged 20–29 years), and lower education (APR = 1.12; 95%CI = 1.06–1.19, compared to those with education beyond high school), were associated with increased likelihood of daily home tobacco use. Conversely, living with adult household members or child/children (APR = 0.92; 95%CI = 0.85–0.997 and APR = 0.83; 95%CI = 0.75–0.91, compared to those living alone) and being a current drinker (APR = 0.90; 95%CI = 0.84–0.96, compared to non-current/never drinkers) were associated with lower likelihoods of daily home tobacco use.

**Table 3:** Prevalence and ratios of tobacco product use in the home among current tobacco product users

	**Distribution**	**Daily use in the home**		**≥Monthly use in the home**	

	**N (%)**	**% (SE)**	**APR (95%CI)**	**% (SE)**	**APR**
Overall	5818 (100.0%)	49.8 (1.0)	-	67.1 (0.9)	-
Tobacco use status					
Combustible tobacco only	2830 (47.9%)	43.2 (1.4)	Ref.	51.5 (1.4)	Ref.
HTP only	1175 (21.1%)	69.5 (1.8)	**1.54 (1.43–1.67)**	79.2 (1.6)	**1.43 (1.34–1.51)**
Dual use	1813 (31.0%)	46.7 (1.7)	**1.10 (1.01–1.20)**	82.9 (1.2)	**1.45 (1.37–1.53)**
Home tobacco use rule					
No tobacco allowed	1622 (26.2%)	13.9 (1.2)	Ref.	25.0 (1.5)	Ref.
Only HTPs allowed	893 (16.7%)	65.9 (2.3)	**4.02 (3.33–4.86)**	89.5 (1.4)	**2.98 (2.63–3.38)**
Only combustible tobacco allowed	127 (2.0%)	42.2 (6.4)	**3.02 (2.19–4.18)**	83.8 (4.8)	**2.89 (2.45–3.41)**
Any tobacco allowed	2404 (40.2%)	70.6 (1.4)	**4.57 (3.83–5.46)**	87.1 (1.0)	**3.22 (2.85–3.63)**
Don’t know/Not applicable	772 (14.8%)	39.9 (2.6)	**2.62 (2.12–3.25)**	59.7 (2.6)	**2.15 (1.86–2.49)**
Workplace tobacco use policy					
No tobacco allowed	1498 (23.7%)	40.2 (1.8)	Ref.	68.2 (1.7)	Ref.
Allowed, outdoor only	1759 (32.1%)	47.2 (1.7)	1.05 (0.95–1.15)	60.4 (1.7)	**0.89 (0.83–0.94)**
Allowed, in designated indoor spaces only	936 (16.4%)	54.6 (2.4)	**1.17 (1.06–1.31)**	72.5 (2.0)	0.99 (0.93–1.05)
Allowed, anywhere	263 (4.6%)	69.0 (3.8)	**1.28 (1.13–1.45)**	79.3 (3.3)	1.02 (0.94–1.11)
Don’t know/Not applicable	1362 (23.3%)	56.1 (2.0)	**1.24 (1.12–1.38)**	68.9 (1.8)	1.02 (0.95–1.09)
Sex					
Man	4126 (72.3%)	53.6 (1.9)	Ref.	67.1 (1.0)	Ref.
Woman	1692 (27.7%)	48.4 (1.1)	1.04 (0.97–1.11)	67.2 (1.8)	0.99 (0.94–1.03)
Age, years					
16–19	52 (0.8%)	16.0 (7.3)	0.52 (0.20–1.37)	66.4 (10.1)	0.95 (0.69–1.32)
20–29	934 (16.4%)	34.1 (2.4)	Ref.	70.2 (2.4)	Ref.
30–39	1016 (20.8%)	43.7 (2.1)	**1.33 (1.14–1.56)**	64.4 (2.1)	1.02 (0.95–1.10)
40–49	1291 (24.1%)	53.1 (1.9)	**1.59 (1.37–1.84)**	65.7 (1.8)	1.06 (0.99–1.13)
50–59	1126 (17.2%)	59.8 (1.9)	**1.64 (1.42–1.90)**	68.0 (1.8)	1.06 (0.99–1.14)
60–74	1399 (20.7%)	57.9 (2.0)	**1.55 (1.33–1.81)**	68.4 (1.9)	1.07 (0.998–1.16)
Education					
High school or below	1916 (61.4%)	53.9 (1.4)	**1.12 (1.06–1.19)**	69.7 (1.3)	**1.07 (1.02–1.11)**
Beyond high school	3866 (38.6%)	43.3 (1.0)	Ref.	62.6 (1.0)	Ref.
Home ownership					
No	2131 (28.1%)	52.1 (1.5)	Ref.	69.7 (1.4)	Ref.
Yes	3687 (71.9%)	49.0 (1.2)	1.03 (0.96–1.11)	66.1 (1.1)	0.98 (0.94–1.03)
Household members					
Alone	1481 (19.4%)	57.3 (1.9)	Ref.	78.7 (1.5)	Ref.
Adults only	2581 (45.4%)	53.8 (1.4)	**0.92 (0.85–0.997)**	67.5 (1.3)	**0.89 (0.85–0.94)**
Child/Children present	1756 (35.2%)	40.6 (1.6)	**0.83 (0.75–0.91)**	60.2 (1.6)	**0.85 (0.80–0.90)**
Alcohol use					
Non-current/never	1671 (31.2%)	56.7 (1.8)	Ref.	68.4 (1.6)	Ref.
Current	4147 (68.8%)	46.7 (1.1)	**0.90 (0.84–0.96)**	66.5 (1.0)	0.97 (0.93–1.02)

**Abbreviation:** APR = adjusted prevalence ratio, CI = confidence interval, HTP = heated tobacco product, Ref. = reference, SE = standard error.

**Note:** Data were weighted to address the selectivity of the internet-based sample using a nationally representative sample of Japanese population. Respondents who had not stayed in their homes in the past month were excluded from the analysis. **Bold** type indicates statistical significance (p < 0.05).

1. Combustible tobacco products assessed in this study included cigarettes (manufactured and roll-your-own cigarettes), little cigars, pipes, and water pipes

2. Heated tobacco products (HTPs) assessed in this study included Ploom Tech, Ploom S, Ploom X, IQOS, glo, and lil HYBRID

Overall, 67.1% of participants reported using tobacco products in their homes at least once a month, with higher percentages among exclusive HTP users (APR = 1.43; 95%CI = 1.34–1.51) and dual users (APR = 1.45; 95%CI = 1.37–1.53) compared to exclusive combustible tobacco smokers. Similarly, ≥monthly home tobacco use was more likely among those with partial or no tobacco-free home rules, with the highest likelihood observed among those with no tobacco-free home rules (APR = 3.22; 95%CI = 2.85–3.63), followed by those allowing HTP use (APR = 2.98; 95%CI = 2.63–3.38) and those allowing combustible tobacco smoking (APR = 2.89; 95%CI = 2.45–3.41), compared to those with complete tobacco-free home rules. Workplace tobacco-free policy was not significantly associated with ≥monthly home tobacco use except for those with policies allowing outdoor tobacco use, which reported a lower likelihood (APR = 0.89; 95%CI = 0.83–0.94, compared to complete workplace tobacco-free policies). Lower education (APR = 1.07; 95%CI = 1.02–1.11, compared to education beyond high school) and the presence of adult household members or child/children (APR = 0.89; 95%CI = 0.85–0.94 and APR = 0.85; 95%CI = 0.80–0.90, compared to those living alone) were significantly associated with ≥monthly home tobacco use, while age and drinking habits did not exhibit significant associations.

## Discussion

In 2023, the prevalence of current tobacco product use in Japan remained consistent with previous years [1, 2]. Among the various tobacco products studied, cigarettes were the most commonly used, with a prevalence of 18.9%, followed by HTPs with a prevalence of 12.4%. A noteworthy proportion of current HTP users (52.2%) and combustible tobacco smokers (38.2%) reported daily usage of their respective products within their homes. This usage varied based on factors including the adoption of tobacco-free rules at home or workplace, age, education, drinking habit, and the presence of adult or child household members. These findings emphasize the need to assess and mitigate the use of tobacco products within the home, including HTPs, and the involuntary exposure to secondhand emissions that could result from it. It is crucial to explore strategies that discourage tobacco use in private spaces to contribute to the broader objective of reducing tobacco-related health risks.

The prevalence of current tobacco product usage within the home was notably high, highlighting the extensive nature of tobacco consumption within domestic settings. It is particularly concerning that even respondents with cohabitants exhibited significant daily usage rates. Even among those living with children, daily home usage was reported by 45.6% of current HTP users and 26.1% of combustible tobacco smokers. Additionally, our study uncovered that merely 46.7% of respondents adopted a comprehensive tobacco-free rule for their homes, which is considerably lower than comparable figures in other countries [18–21]. For instance, in the United States, the adoption rate of smoke-free home rules has made substantial progress, exceeding 90% in 2018–2019 due to coordinated efforts by federal, state, and local initiatives aimed at educating the public about the risks associated with SHS [18, 22]. Notably, our investigation also revealed that those who asserted having comprehensive tobacco-free home rules did not consistently adhere to them. Given that both residences and workplaces are primary sources of exposure to SHS and secondhand aerosol from HTPs in Japan [10, 11], these findings underscore the urgent requirement for targeted interventions that address potential exposure risks for vulnerable populations.

Regarding tobacco use policies in workplaces, respondents lacking complete tobacco-free policies were more prone to report daily tobacco product use in the home. Earlier research indicates that individuals working in environments with robust tobacco-free policies are more inclined to reduce tobacco usage [12, 13], which could lead to decreased daily tobacco consumption at home. Prior to the introduction of the revised Health Promotion Act in 2020, there were concerns that implementing a complete workplace tobacco-use ban might lead to increased tobacco use at home. However, this study revealed that such concerns were unfounded. Ensuring the full enforcement of tobacco-free regulations in workplaces is imperative. While the revised Health Promotion Act serves as a safeguard within workplaces and other public places, extending similar regulations to private spaces requires careful evaluation in terms of feasibility and public acceptance. Health education campaigns could potentially play a pivotal role in cultivating a culture of tobacco-free homes.

Furthermore, the notably high prevalence of home use among HTP users warrants attention. This observation might be indicative of the health-conscious image that HTPs have strategically conveyed [4–6]. The concept of “reduced-exposure” might make users feel less concerned about potential indoor harm, contributing to the observed usage patterns. The intensified marketing of HTPs during the COVID-19 pandemic, encouraging their consumption at home [7, 8], highlights the industry’s ability to adapt and capitalize on evolving circumstances. This approach implies a deliberate effort to promote tobacco use in environments seen as safer, which could potentially undermine public health efforts to denormalize tobacco use and reduce involuntary exposure to tobacco emissions in the home. It is crucial not to overlook the potential health risks of HTPs to people living with HTP users, considering the significant levels of pollutants and carcinogenic compounds present in secondhand aerosol from HTPs [23, 24].

Aligned with previous studies, this study revealed demographic and socioeconomic disparities in home tobacco use [25, 26]. The lower likelihood of daily tobacco use at home among younger generations can be attributed to various factors. The living environment may play a role; younger individuals living independently may frequently encounter tobacco-free policies in apartments. Those residing with their families might also face restrictions on home tobacco use imposed by family members, such as parents, in shared living spaces. Moreover, tobacco use behaviors in younger generations are notably influenced by social factors. Active social lives with their peers among the younger demographic provide more opportunities for tobacco use in social settings, potentially contributing to lower daily tobacco use at home. The increased likelihood of daily tobacco use in the home among less educated groups may stem from disparities in health literacy across educational levels, highlighting the necessity for targeted educational interventions. Notably, individuals who are current drinkers displayed a lower inclination to use HTPs on a daily basis within their homes. This could be attributed to the availability of tobacco use opportunities in social settings where such behavior is allowed, potentially reducing the need for consumption at home. These nuances underscore the intricate interplay between behaviors and environments that shape tobacco use patterns.

The present study had certain limitations. Since our study sample was acquired through Internet-based recruitment, the findings might not be applicable to individuals who have limited Internet access or literacy. Nevertheless, it is worth noting that Internet penetration in Japan exceeded 90% as of 2021 [27], and we employed weighted data to counterbalance any variations in socioeconomic and demographic attributes between the participants of our Internet-based survey and a nationally representative populace. As such, the potential for selection bias in this study is deemed to be minimal. Moreover, the reliance on self-reported data could potentially introduce reporting errors (such as recall inaccuracies or confusion between heated tobacco product use and combustible tobacco smoking), despite our efforts to mitigate such potential inaccuracies by employing distinct questions for each type of tobacco product.

## Conclusion

A significant proportion of current tobacco product users reported using tobacco at home, particularly among HTP users, those without complete tobacco-free rules at home or workplaces, older individuals, and those with lower education. Along with the full enforcement of tobacco-free regulations in public places, targeted educational strategies should be explored to discourage tobacco use within private spaces.

## Abbreviations

APR: Adjusted prevalence ratio
CI: Confidence interval
HTP: Heated tobacco product
IPW: Inverse probability weight
JASTIS: Japan Society and New Tobacco Internet Survey
SHS: Secondhand smoke
WHO: World Health Organization

## Supplementary information

**Additional file 1:** **Supplementary Table 1.** Prevalence of current tobacco product use by sex in Japan, 2023.

## Declarations

### Availability of data and material

The data are not publicly available due to privacy restrictions and only available on request to the corresponding author at tabuchitak@gmail.com.
