# Supplementary material for: Tobacco usage in the home: a cross-sectional analysis of heated tobacco product (HTP) use and combustible tobacco smoking in Japan, 2023
Source: Environ Health Prev Med. 2024 Mar 5;29:11. doi: 10.1265/ehpm.23-00292 (PMC10937248; doi:10.1265/ehpm.23-00292)
Supplement: Supplementary file 1 — Additional file 1: Supplementary Table 1. Prevalence of current tobacco product use by sex in Japan, 2023. [file ehpm-29-011-s001.docx]

**Supplementary Table 1.** Prevalence of current tobacco product use by sex in Japan, 2023

**Men**

|  |  | Prevalence | | | | |
| --- | --- | --- | --- | --- | --- | --- |
|  | Distribution | Heated tobacco products | Cigarettes | Non-cigarette combustible tobacco products | Dual (combustible + heated tobacco) use | Any (1+) tobacco product |
|  | N (%) | % (SE) | % (SE) | % (SE) | % (SE) | % (SE) |
| Overall | 14482 (100%) | 18.9 (0.5) | 27.6 (0.6) | 5.4 (0.3) | 11.6 (0.4) | 35.8 (0.6) |
| Home tobacco use rule |  |  |  |  |  |  |
| No tobacco allowed | 7100 (45.6%) | 9.3 (0.5) | 18.3 (0.7) | 2.6 (0.3) | 4.8 (0.4) | 23.2 (0.8) |
| Only HTPs allowed | 895 (6.9%) | 71.6 (2.1) | 45.4 (2.3) | 14.9 (1.7) | 37.8 (2.3) | 80.2 (1.9) |
| Only combustible tobacco allowed | 217 (1.6%) | 37.6 (4.6) | 40.6 (4.6) | 19.1 (3.4) | 26.3 (4.1) | 53.9 (4.7) |
| Any tobacco allowed | 2651 (19.0%) | 34.0 (1.3) | 56.5 (1.4) | 9.0 (0.8) | 23.4 (1.2) | 68.3 (1.3) |
| Don't know/Not applicable | 3619 (26.9%) | 10.2 (0.8) | 17.9 (1.0) | 4.6 (0.5) | 7.1 (0.7) | 21.7 (1.0) |
| Workplace tobacco use policy |  |  |  |  |  |  |
| No tobacco allowed | 4055 (24.9%) | 19.7 (0.9) | 27.0 (1.1) | 8.4 (0.7) | 14.1 (0.8) | 33.1 (1.1) |
| Allowed, outdoor only | 4184 (29.8%) | 21.5 (0.9) | 29.2 (1.0) | 3.9 (0.4) | 10.9 (0.7) | 40.7 (1.1) |
| Allowed, in designated indoor spaces only | 1815 (12.8%) | 27.1 (1.5) | 36.4 (1.7) | 6.8 (0.9) | 16.7 (1.3) | 47.7 (1.7) |
| Allowed, anywhere | 382 (3.3%) | 27.1 (3.1) | 41.0 (3.5) | 5.3 (1.2) | 15.8 (2.5) | 54.1 (3.7) |
| Don't know/Not applicable | 4046 (29.2%) | 11.2 (0.7) | 21.2 (1.0) | 3.9 (0.4) | 7.3 (0.6) | 25.8 (1.0) |
| Age, years |  |  |  |  |  |  |
| 16-19 | 309 (2.1%) | 10.6 (3.1) | 9.9 (2.8) | 6.1 (2.5) | 8.3 (2.7) | 12.4 (3.2) |
| 20-29 | 2678 (18.6%) | 21.6 (1.3) | 25.0 (1.4) | 10.3 (0.9) | 16.8 (1.2) | 30.9 (1.4) |
| 30-39 | 2820 (19.6%) | 25.3 (1.2) | 28.2 (1.2) | 6.3 (0.6) | 15.2 (1.0) | 39.0 (1.3) |
| 40-49 | 2740 (19.1%) | 24.4 (1.2) | 32.0 (1.2) | 5.0 (0.6) | 13.2 (0.9) | 43.8 (1.3) |
| 50-59 | 2259 (15.7%) | 16.4 (1.0) | 30.1 (1.3) | 2.8 (0.4) | 8.5 (0.7) | 38.8 (1.3) |
| 60-74 | 3676 (24.9%) | 10.2 (0.8) | 25.8 (1.2) | 3.0 (0.4) | 5.7 (0.6) | 30.9 (1.2) |
| Home ownership |  |  |  |  |  |  |
| No | 5070 (26.4%) | 19.7 (0.8) | 27.7 (0.9) | 7.1 (0.5) | 12.2 (0.6) | 36.2 (1.0) |
| Yes | 9412 (73.6%) | 18.7 (0.6) | 27.6 (0.7) | 4.8 (0.3) | 11.3 (0.5) | 35.7 (0.7) |
| Household members |  |  |  |  |  |  |
| Alone | 3786 (20.4%) | 19.4 (1.0) | 28.5 (1.1) | 7.5 (0.6) | 13 (0.9) | 35.9 (1.2) |
| Adults only | 6825 (49.8%) | 14.7 (0.6) | 25.3 (0.8) | 4.2 (0.4) | 8.5 (0.5) | 32.3 (0.8) |
| Child/Children present | 3871 (29.8%) | 25.7 (1.0) | 30.9 (1.0) | 6.1 (0.5) | 15.7 (0.8) | 41.5 (1.1) |
| Education |  |  |  |  |  |  |
| High school or below | 3583 (55.8%) | 18.9 (0.8) | 30.7 (0.9) | 5.1 (0.4) | 11.6 (0.7) | 38.7 (1.0) |
| Beyond high school | 10763 (44.2%) | 18.9 (0.5) | 24.0 (0.5) | 5.7 (0.3) | 11.5 (0.4) | 32.5 (0.5) |
| Alcohol use |  |  |  |  |  |  |
| Non-current/never | 5151 (36.9%) | 12.4 (0.7) | 20.4 (0.9) | 2.5 (0.3) | 7.1 (0.6) | 26.3 (0.9) |
| Current | 9331 (63.1%) | 22.7 (0.6) | 31.8 (0.7) | 7.2 (0.4) | 14.2 (0.5) | 41.3 (0.7) |

**Women**

|  |  | Prevalence | | | | |
| --- | --- | --- | --- | --- | --- | --- |
|  | Distribution | Heated tobacco products | Cigarettes | Non-cigarette combustible tobacco products | Dual (combustible + heated tobacco) use | Any (1+) tobacco product |
|  | N (%) | % (SE) | % (SE) | % (SE) | % (SE) | % (SE) |
| Overall | 14872 (100.0%) | 6.1 (0.3) | 10.4 (0.4) | 1.9 (0.2) | 3.4 (0.2) | 13.6 (0.4) |
| Home tobacco use rule |  |  |  |  |  |  |
| No tobacco allowed | 7612 (47.6%) | 2.0 (0.2) | 6.2 (0.4) | 1.1 (0.2) | 1.4 (0.2) | 7.1 (0.5) |
| Only HTPs allowed | 557 (4.5%) | 40.9 (3.3) | 21.0 (2.7) | 5.1 (1.5) | 15.1 (2.3) | 47.2 (3.3) |
| Only combustible tobacco allowed | 106 (0.6%) | 26.1 (5.9) | 26.2 (5.9) | 22.4 (5.7) | 24.3 (5.8) | 31.7 (6.2) |
| Any tobacco allowed | 2122 (14.6%) | 14.5 (1.2) | 29.6 (1.5) | 3.3 (0.5) | 9.2 (1.0) | 35.8 (1.6) |
| Don't know/Not applicable | 4475 (32.6%) | 3.0 (0.4) | 6.2 (0.6) | 1.5 (0.3) | 1.8 (0.3) | 8.2 (0.6) |
| Workplace tobacco use policy |  |  |  |  |  |  |
| No tobacco allowed | 4228 (25.8%) | 6.0 (0.6) | 9.6 (0.7) | 2.2 (0.3) | 3.5 (0.5) | 13.0 (0.8) |
| Allowed, outdoor only | 2758 (19.4%) | 7.6 (0.7) | 14.1 (1.0) | 2.0 (0.4) | 4.3 (0.5) | 17.8 (1.1) |
| Allowed, in designated indoor spaces only | 1164 (6.9%) | 15.2 (1.8) | 15.7 (1.7) | 5.1 (1.2) | 7.9 (1.3) | 23.7 (2.1) |
| Allowed, anywhere | 198 (1.6%) | 11.9 (2.7) | 18.8 (3.7) | 1.1 (0.6) | 4.8 (1.6) | 25.9 (4.3) |
| Don't know/Not applicable | 6524 (46.3%) | 3.9 (0.4) | 8.2 (0.5) | 1.1 (0.2) | 2.2 (0.3) | 10.3 (0.6) |
| Age, years |  |  |  |  |  |  |
| 16-19 | 413 (2.8%) | 4.3 (1.1) | 4.4 (1.2) | 3.5 (1.0) | 3.4 (1.0) | 6.7 (1.4) |
| 20-29 | 2809 (18.9%) | 8.1 (0.9) | 9.0 (0.9) | 4.0 (0.6) | 5.6 (0.7) | 13.0 (1.1) |
| 30-39 | 2932 (19.7%) | 7.6 (0.8) | 9.7 (0.9) | 1.8 (0.4) | 3.9 (0.6) | 14.1 (1.1) |
| 40-49 | 2745 (18.5%) | 8.4 (0.8) | 14.7 (1.0) | 1.9 (0.5) | 4.4 (0.6) | 19.1 (1.1) |
| 50-59 | 2314 (15.6%) | 5.0 (0.6) | 11.3 (0.9) | 1.2 (0.3) | 2.6 (0.4) | 14.0 (1.0) |
| 60-74 | 3659 (24.5%) | 2.3 (0.4) | 8.8 (0.7) | 0.4 (0.2) | 1.1 (0.2) | 10.2 (0.8) |
| Home ownership |  |  |  |  |  |  |
| No | 5218 (26.3%) | 7.7 (0.5) | 12.8 (0.8) | 2.3 (0.3) | 4.5 (0.4) | 16.8 (0.8) |
| Yes | 9654 (73.7%) | 5.5 (0.4) | 9.5 (0.5) | 1.7 (0.2) | 3.0 (0.3) | 12.5 (0.5) |
| Household members |  |  |  |  |  |  |
| Alone | 2861 (15.2%) | 6.2 (0.8) | 10.8 (0.9) | 2.4 (0.6) | 3.7 (0.7) | 14.2 (1.0) |
| Adults only | 7088 (51.4%) | 4.8 (0.4) | 9.8 (0.5) | 1.2 (0.2) | 2.9 (0.3) | 12 (0.6) |
| Child/Children present | 4923 (33.4%) | 8 (0.6) | 11.1 (0.7) | 2.5 (0.4) | 4.1 (0.4) | 15.8 (0.8) |
| Education |  |  |  |  |  |  |
| High school or below | 4263 (52.0%) | 7.3 (0.5) | 12.6 (0.7) | 1.8 (0.3) | 3.9 (0.4) | 16.4 (0.8) |
| Beyond high school | 10513 (48.0%) | 4.8 (0.3) | 8.0 (0.4) | 2.0 (0.2) | 2.8 (0.2) | 10.7 (0.4) |
| Alcohol use |  |  |  |  |  |  |
| Non-current/never | 7959 (55.9%) | 4.1 (0.3) | 8.0 (0.5) | 1.0 (0.2) | 1.9 (0.2) | 10.6 (0.6) |
| Current | 6913 (44.1%) | 8.6 (0.5) | 13.4 (0.6) | 2.9 (0.3) | 5.4 (0.4) | 17.5 (0.7) |

**Abbreviation:** HTP=heated tobacco product, SE=standard error.

**Note:** Data were weighted to address the selectivity of the internet-based sample using a nationally representative sample of Japanese population.

1. Cigarettes assessed in this study included manufactured cigarettes and roll-your-own cigarettes
2. Non-cigarette combustible tobacco products assessed in this study included little cigars, pipes, and water pipes
3. Heated tobacco products (HTPs) assessed in this study included Ploom Tech, Ploom S, Ploom X, IQOS, glo, and lil HYBRID
